# Lysosomal metabolism in pancreatic cancer and its crosstalk with host physiology

**DOI:** 10.1101/gad.353702.126

**Published:** 2026-10-01

**Authors:** Caleb Cheng, Rüya Pakkan, Arul M. Chinnaiyan, Costas A. Lyssiotis

**Affiliations:** 1 Michigan Center for Translational Pathology, University of Michigan, Ann Arbor, Michigan 48109, USA;; 2 Medical Scientist Training Program, University of Michigan, Ann Arbor, Michigan 48109, USA;; 3 Cellular and Molecular Biology Program, University of Michigan, Ann Arbor, Michigan 48109, USA;; 4 Department of Pathology, University of Michigan, Ann Arbor, Michigan 48109, USA;; 5 Rogel Cancer Center, University of Michigan, Ann Arbor, Michigan 48109, USA;; 6 Howard Hughes Medical Institute, University of Michigan, Ann Arbor, Michigan 48109, USA;; 7 Department of Urology, University of Michigan, Ann Arbor, Michigan 48109, USA;; 8 Department of Molecular and Integrative Physiology, University of Michigan, Ann Arbor, Michigan 48109, USA;; 9 Department of Internal Medicine, Division of Gastroenterology, University of Michigan, Ann Arbor, Michigan 48109, USA

**Keywords:** lysosome metabolism, pancreatic cancer, tumor host metabolism

## Abstract

Pancreatic ductal adenocarcinoma (PDAC) grows within a highly fibrotic, pressurized microenvironment that collapses vasculature and restricts delivery of oxygen and circulating nutrients. To survive this metabolic stress, PDAC cells activate lysosome-centered nutrient acquisition and recycling programs, including macroautophagy, RAS-driven macropinocytosis, and receptor-mediated endocytosis, that traffic intracellular and extracellular cargo to lysosomes for degradation and metabolite export. These pathways are reinforced by oncogenic signaling and MiT/TFE-dependent lysosomal biogenesis, and they support core outputs of tumor metabolism such as iron bioavailability, amino acid and nucleotide pools, lipid homeostasis, and immune evasion. Lysosomal programs in nonmalignant compartments (fibroblasts, stellate cells, and immune cells) further shape nutrient exchange, matrix production, and whole-body metabolism, positioning the lysosome as a key node at the tumor–host interface. Although genetic and pharmacologic blockade of autophagy/lysosome function can produce potent antitumor effects in preclinical models, clinical trials with lysosomotropic agents have shown limited benefit, highlighting challenges in target engagement, biomarkers, and rational combination strategies. Here we review current tools and concepts for interrogating lysosomal flux in PDAC, integrate emerging insights from systemic metabolism and dietary interventions, and outline therapeutic opportunities for more effectively exploiting lysosome dependence in pancreatic cancer.

## Abstract (precis)

In this Review, Cheng et al. discuss how pancreatic cancer cells adapt to metabolic stress in the tumor microenvironment through the activation of lysosomal nutrient recycling and acquisition programs. Lysosomal pathways help maintain various tumor cell-autonomous and -nonautonomous metabolic programs, positioning lysosomal flux as an important, and potentially targetable, adaptive mechanism at the tumor-host interface.

## Pancreatic cancer cells employ lysosomal nutrient recycling and acquisition pathways to adapt to a nutrient deregulated tumor microenvironment

Pancreatic ductal adenocarcinoma (PDAC) tumors contain relatively few carcinoma cells inside a densely packed stroma, populated by immune cells, fibroblasts, and extracellular matrix, notably hyaluronic acid (Chu et al. 2007; Provenzano et al. 2012). The extracellular matrix retains water, which leads to interstitial pressures that exceed 10 times those observed in the normal pancreas (Provenzano et al. 2012; Chauhan et al. 2014). Under this pressure, vasculature collapses, leading to decreased access to circulation (Olive et al. 2009; DuFort et al. 2016). This tumor physiology explains the findings that the nutritional microenvironment of PDAC tumors are dramatically disrupted compared to surrounding normal pancreatic tissue or plasma (Kamphorst et al. 2015; Sullivan et al. 2019). The disrupted nutritional microenvironment, in collaboration with the nearly ubiquitous genomic mutations in KRAS, result in prominent metabolic reprogramming of PDAC cells (De Santis et al. 2024)—a hallmark of the disease.

To compensate for the limited access to blood-derived nutrients, PDAC cells adapt to become expert nutrient scavengers and recyclers. Notably, RAS pathway activation promotes macropinocytosis, an actin-driven, process of “cell drinking” that allows PDAC cells to acquire and degrade extracellular material to obtain amino acids (Bar-Sagi and Feramisco 1986; Commisso et al. 2013; Davidson et al. 2017). PDAC cells also employ receptor-mediated endocytosis to acquire specific nutrients such as lipids through low density lipoprotein receptor (*LDLR*) to support lipid metabolism (Guillaumond et al. 2015) or iron through transferrin receptor (*TFRC*) to support iron metabolism (Jeong et al. 2016). Finally, PDAC cells are well-known to exhibit a high-level of macroautophagy (referred to here as autophagy), allowing for turnover of intracellular proteins and organelles to support cellular metabolism (Yang et al. 2011; Yamamoto et al. 2023). These scavenging and recycling pathways coalesce on the lysosome, which enables degradation of nutrients into base macromolecules that are subsequently exported through lysosomal channels for use in bioenergetics or biosynthesis (Fig. 1). To support lysosomal demand, the microphthalmia/transcription factor E (MiT/TFE3) transcription factors, which includes MiT, TFEB, TFE3, and TFEC and regulate genes involved in lysosomal biogenesis and function, are highly upregulated in PDAC cells compared to normal pancreas and even across other solid tumor cells (Perera et al. 2015). Altogether, this highlights the importance of lysosome-dependent nutrient acquisition/recycling pathways in PDAC cellular metabolism.

**Figure 1.**
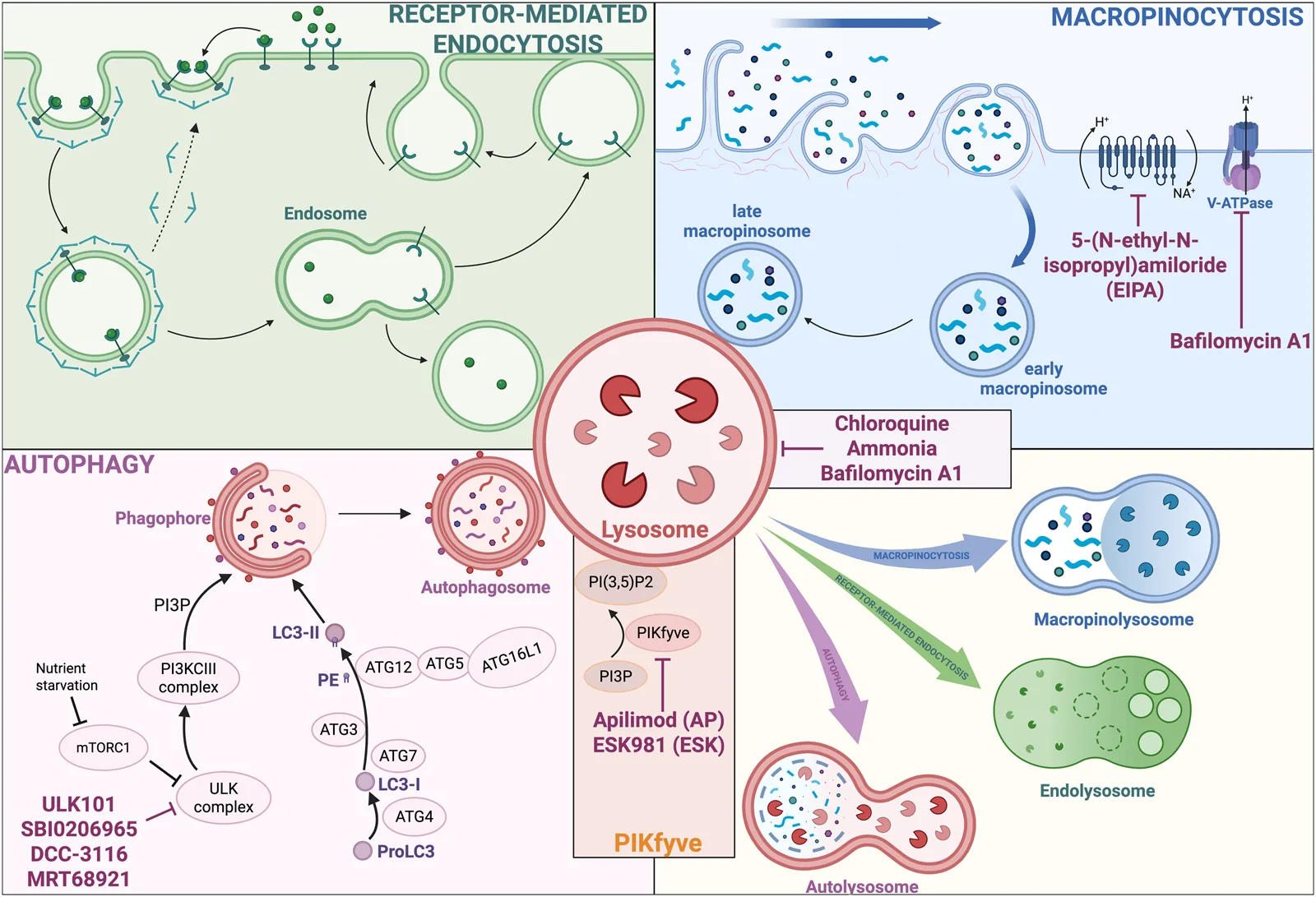
Lysosome-centric view of nutrient acquisition/recycling programs: receptor-mediated endocytosis, macropinocytosis, and autophagy. Inhibitors prominently featured in pancreatic cancer studies, along with their targets, are highlighted.

## Current approaches to studying lysosomal nutrient recycling and acquisition programs in pancreatic cancer

PDAC cells grown in nutrient replete conditions in vitro continue to exhibit an elevated usage of these lysosomal nutrient scavenging and recycling pathways (Yang et al. 2011) due to rewired regulation of the MiTF/TFE factors (Perera et al. 2015). This unlocks the ability to study these pathways using genetic and pharmacological methods, as well as to study the contents inside the vesicles being trafficked to the lysosomes. As depicted in Figure 1, endosomes, autophagosomes, and macropinosomes represent the core vesicular trafficking mechanisms to bring intracellular and extracellular nutrients to the lysosome. In the section that follows, we describe the molecular machinery involved in regulating and executing these processes with the lens of identifying genetic targets and associated pharmacologic compounds that have been utilized to manipulate these pathways in PDAC or other models. We will then briefly highlight molecular tools and assays that can or have been used to study these pathways. Throughout, we will summarize conclusions that can be drawn from studies that employ these techniques and technologies, and any caveats that we perceive as important to consider.

### Genetic targets involved in lysosome-dependent nutrient recycling and acquisition pathways

Autophagy is an evolutionarily conserved cellular recycling process that is initiated when cells are energy stressed (e.g., in low nutrient conditions) and allows for degradation of superfluous or damaged organelles, proteins, or other structures and recycling them into base macromolecules useful for bioenergetics or biosynthesis. This process is thus tightly regulated and linked to energy and nutrient states by mammalian Target of Rapamycin (mTORC1) and AMP-activated protein kinase (AMPK). In nutrient (i.e., amino acid) replete conditions, Rag GTPases traffic mTORC1 to lysosomal membranes where it then phosphorylates (and thus inhibits) ATG13 and ULK1, two important proteins in the ULK1 complex (involving ULK1/2, FIP200, ATG13, and ATG101) that is responsible for autophagy initiation (Zoncu et al. 2011a,b). Under starved conditions, this phosphorylation is removed and the complex can thus form (Fig. 1, bottom left). AMPK has also been shown to both suppress mTORC1 as well as directly phosphorylate ULK1, enabling it to both indirectly and directly activate autophagy under energy starvation (Egan et al. 2011; Kim et al. 2011).

Activation of the ULK1 complex is the first step of the autophagic pathway, which occurs at the endoplasmic reticulum. This results in the formation of a budding double-membraned structure termed the phagophore. This is coordinated with the class III PI3K complex (involving Beclin-1, VPS14, VPS34, and ATG 14L), which produces phosphpatidylinositol-3-phosphate (PI3P) that signals the recruitment of the two ubiquitin-like systems: the ATG8 (LC3) system, mediated by ATG7 and ATG3, and the ATG5/12/16L1 system. Both signals are required for the conjugation of LC3 to phosphatidylethanolamine (PE). The conjugation of LC3 to PE (conversion of LC3-I to LC3-II) is then required for its incorporation in the elongating phagophore and thus its critical roles tethering cargo for degradation and maturation of the autophagosome (Fig. 1, bottom left; Debnath et al. 2023).

Genetic perturbation modalities allow for interrogation of the roles of specific proteins in the autophagy machinery. Here, we will list the methods that have been employed and describe the collective conclusions that can be surmised based on the reported results. Core autophagy related genes ATG5 and ATG7 have been knocked out of PDAC cells using CRISPR-mediated gene editing in vitro (Bao et al. 2023) as well as in vivo (Yang et al. 2014; Zhu et al. 2021). Similarly, ATG3, ATG5, and ATG7 have all been targeted with siRNA or shRNAs (Yang et al. 2011; Mukhopadhyay et al. 2021). However, given that cancer cells can adapt to loss of autophagy, an acute, live-cell imaging CRISPR-ribonucleoprotein (CRISPR-RNP) assay targeting several genes in the pathway was developed to assess immediate effects of autophagy inhibition (Towers et al. 2019). Finally, a doxycycline-inducible dominant negative ATG4B mutant (Fujita et al. 2008) model has been used to toggle autophagic flux both in vitro and in vivo (Yang et al. 2018). Importantly, although this review is focused on canonical macroautophagy, all the above methods should also inhibit the noncanonical autophagic pathway, conjugation of ATG8 to single membranes (CASM) (Durgan and Florey 2022) but not chaperone-mediated autophagy (Kaushik and Cuervo 2018) or microautophagy (Yamamoto et al. 2023).

In general, data generated using genetic perturbations of autophagy have suggested that autophagy is dispensable in optimal growth conditions but necessary in situations of cellular stress. Specifically, multiple studies employing CRISPR or the overexpression of the dominant negative ATG4B mutant have suggested that autophagy is dispensable in vitro in nutrient replete conditions (White 2015; Eng et al. 2016; Yang et al. 2018; Zhu et al. 2021) or in vivo in immune-deficient mice (Yamamoto et al. 2020; Zhu et al. 2021). Similarly, while siRNA or shRNA-mediated knockdown of ATG5 or ATG7 led to decreased colony number in colony growth assays (Yang et al. 2011; Mukhopadhyay et al. 2021, 2023), to our knowledge, there has not been a report demonstrating that knockdown/knockout of any ATGs lead to blunted cell growth in 2D under nutrient-replete growth conditions. These data suggest that PDAC cells are conditionally dependent on autophagy in nutrient-deficient environments. It is also important to note that these studies assessed the effects of tumor-specific genetic perturbation of autophagy, contrasting pharmacological inhibition in which both tumor and host cell autophagy are inhibited. This may play a large and underappreciated role as inhibition of host autophagy has been shown to strongly impact tumor growth (Sousa et al. 2016; Poillet-Perez et al. 2018).

Receptor-mediated endocytosis, also known as clathrin-mediated endocytosis, is a process through which cells can uptake metabolites, proteins, or other molecules specified by a receptor. Binding of the ligand (e.g., nutrient-loaded cargo) to the receptor recruits adaptor proteins and clathrin, leading to invagination and eventual cleavage of a clathrin-coated pit into a vesicle. This vesicle can then be fused with an endosome, which leads to fusion with the lysosome or recycling back to the plasma membrane (Fig. 1, top left). Relevant to PDAC, Low Density Lipoprotein Receptor (*LDLR*) is upregulated and can be targeted by genetic silencing to prevent receptor-mediated cholesterol uptake (Guillaumond et al. 2015). Interestingly, regulation of LDL-mediated cholesterol import by PCSK9 was recently found to determine metastatic organ choice in PDAC (Rademaker et al. 2025), further highlighting its important role in PDAC metabolic homeostasis. Similarly, Transferrin Receptor (*TFRC*) is also overexpressed in PDAC cells compared to healthy pancreatic tissue (Ryschich et al. 2004). These two represent well-studied examples of receptor-mediated endocytosis being employed in PDAC cells at an elevated level, contributing to their ability to maintain lipid and iron homeostasis.

Macropinocytosis is an actin-dependent but clathrin- and caveolin-independent mechanism by which cells nonspecifically uptake extracellular material. During initiation, actin polymerization causes the plasma membrane to form protrusions, known as membrane ruffles, which encapsulate extracellular contents in a “cup.” A macropinosome results from the construction and closure of the cup. The macropinosome can then be trafficked to the lysosome for digestion (Fig. 1, top right). In PDAC, macropinocytosis is constitutively active by RAS signaling through RAC1 (Walsh and Bar-Sagi 2001). Knockdown of KRAS and, consequently inhibition of RAC1 (although this was not explicitly shown [Chen et al. 2024]), leads to a decrease in macropinocytosis (Commisso et al. 2013). Additionally, Syndecan 1 (SDC1) was also reported as a critical link between KRAS and RAC1 signaling (Yao et al. 2019), serving as a third viable target for genetic perturbation of macropinocytosis, this crucial KRAS-driven nutrient acquisition pathway for PDAC.

More generally, there also exist a variety of genetic targets to block lysosomal activity (Bonam et al. 2019), ranging from genes involved in lysosome biogenesis such as the MiTF/TFE factors (Perera et al. 2015), surface proteins such as LAMP2(Yang et al. 2011), or specific molecular transport channels such as NPC1 (Davis et al. 2021). Importantly, the MiTF/TFE family of transcription factors was found to be overexpressed and constitutively active, irrespective of MTORC1 signaling, supporting a high level of lysosomal biogenesis and activity. Further, knockdown of these transcription factors decreased tumor growth while overexpression of MITF led to increased tumor growth, indicating the relevance of these pathways to PDAC tumor physiology (Perera et al. 2015). Our laboratory and others have also demonstrated that knockout or knockdown of PIKfyve, the lipid kinase responsible for generating the only cellular source of PI(3,5)P2 and PI5P (Rivero-Ríos and Weisman 2022), blocks lysosomal function (Fig. 1; Bao et al. 2023; DeLiberty et al. 2025). PI(3,5)P2 and PI5P signals are crucial in maintaining ion and pH homeostasis in the lysosome, as well as in the endomembrane system (Rivero-Ríos and Weisman 2022). Thus, perturbing PIKfyve disrupts autophagy and lysosome function by decreasing the levels of these phosphoinositides, likely in part due to decreasing function of ion channel TRPML1 (Krishna et al. 2016). These studies collectively highlight the relevance of lysosomal pathways for PDAC tumor growth.

### Pharmacological agents targeting the lysosome and associated pathways

There is a pervasive misnomer in the literature, where many agents that target the lysosome are inaccurately referred to as “autophagy inhibitors.” While they certainly inhibit autophagy, in part by inhibiting the lysosome, when considering that other pathways converge on the lysosome, our opinion is that it is important to make this distinction. Additionally, when considering the landscape of pharmacological inhibitors of the lysosome or autophagy, it is critical to understand that pharmacological inhibitors can possess and act in part through off (or secondary) targets. Thus, conclusions drawn solely from inhibitor-based studies must be taken with appropriate caution until subsequently validated. Here, we will summarize and attempt to disambiguate autophagy inhibitors, macropinocytosis inhibitors, and lysosome inhibitors that have been used in PDAC or related cancer models. A more comprehensive list of pharmacological agents affecting these pathways can also be found in previous reviews (Gillson et al. 2022).

The most widely used autophagy inhibitors are lysosome inhibitors (Fig. 1). For example, bafilomycin A1 inhibits vacuolar H+ ATPases (V-ATPases), as well as ER-calcium ATPase Ca-P60A/dSERCA, which blocks autophagosome-lysosome fusion and lysosome acidification (Mauvezin and Neufeld 2015). However, as V-ATPases are found on many other organelles, such as the golgi apparatus, secretory vesicles, and even the plasma membrane on certain cell types, bafilomycin A1's effects on cells cannot be completely attributed to its action at the lysosomes (Futai et al. 2019). Additionally, bafilomycin A1 has also been shown to be a potassium ionophore, suggesting it may also impair mitochondrial function independent of its action at V-ATPases (Teplova et al. 2007). Ammonia similarly inhibits lysosomal acidification and thus decreases the activity of degradative enzymes (Seglen et al. 1979). Chloroquine and its derivatives including Lys05 (McAfee et al. 2012) and DQ661 were originally considered to also inhibit autophagy by impacting lysosomal acidity. However, when compared to bafilomycin, it has been shown that chloroquine affects autophagy primarily through decreasing autophagosome-lysosome fusion rather than through affecting lysosomal pH (Mauthe et al. 2018). It has also been suggested that chloroquine may exert its antitumor effects through targeting palmitoyl-protein thioesterase 1 (PPT1) (Rebecca et al. 2019). Of these, only chloroquine has progressed to clinical trials as an autophagy inhibitor; however, chloroquine has also been shown to disturb golgi and endo-lysosomal systems independent of autophagy (Mauthe et al. 2018). Thus, while these inhibitors have been commonly used to represent autophagy inhibition, it is important to note that these inhibitors disrupt more than autophagy specifically, be it the lysosome itself or even nonlysosomal targets.

A multitude of inhibitors have been developed targeting the lipid kinase PIKfyve (Fig. 1). PIKfyve is the only enzyme that can generate phosphatidylinositol 3,5-bisphosphate (PI3,5P2), and it does so by phosphorylating PI3P. While PI(3,5)P2 is most well known for its roles in regulating lysosomal ion and pH as well as lysosomal membrane fission; however, it has also been shown to regulate endosome trafficking as well. Thus, while we believe the dominant cellular effect of PIKfyve inhibition to originate from lysosomal defects, disruption of vesicular trafficking is also affected by PIKfyve inhibition and likely contributes to the cellular phenotypes observed. These inhibitors include apilimod (Cai et al. 2013), APY0201 (Hayakawa et al. 2014), YM201636 (Jefferies et al. 2008), PIK001(Bonolo De Campos et al. 2025), and others (Burke et al. 2023), degraders such as PIK5-12d (Li et al. 2023) and PIK5-33d (Cheng et al. 2025), as well as the multikinase inhibitor ESK981 (Qiao et al. 2021). Apilimod is an extremely useful tool compound given its specificity for PIKfyve (Gayle et al. 2017), but it has poor in vivo pharmacokinetics, which likely contribute to its weak tumor growth control (Ikonomov et al. 2019). ESK981, on the other hand, has potent single-agent efficacy in multiple tumor models and especially PDAC (Qiao et al. 2021; Cheng et al. 2025); however, its identity as a multikinase inhibitor requires contextualization about contributing effects from secondary targets. Notably, ESK981 has known secondary targets, including VEGFR-1, VEGFR-2, TIE-2, FGFR-1, c-SRC, and Aurora A (Hudkins et al. 2012; Pili et al. 2014). Thus, the phenotypes, particularly in vivo, observed by ESK981 must be appropriately caveated without further validation.

Instead, autophagy can be directly and specifically inhibited downstream from ULK1. Indeed, the most common target of autophagy inhibitors is ULK1, with notable inhibitors such as SBI-0206965 (Egan et al. 2015; Bryant et al. 2019), MRT68921 (Petherick et al. 2015; Su et al. 2021), ULK101 (Martin et al. 2018), and DCC3116 (Ghazi et al. 2024) that have been employed in PDAC or other RAS-driven cancer models (Fig. 1). Other inhibitors of ATG4 and other ATGs have also been utilized and have been more comprehensively reviewed previously (Raufi et al. 2021). Finally, autophagy can be induced by inhibiting mTORC1 pharmacologically or by depriving cells of nutrients such as amino acids. However, it is also important to note that, as with most pharmacological agents, secondary targets have been identified for these inhibitors, such as AMPK, the NUAK family SNF1-like kinase 1 (NAUK1), CAMK family members, other others (Dite et al. 2018; Martin et al. 2018; Chen et al. 2020; Knudsen et al. 2020). Thus, orthogonal confirmation using genetic modulation is imperative to confirm effects of autophagy inhibition.

Macropinocytosis is most commonly directly inhibited by 5-(*N*-ethyl-*N*-isopropyl)amiloride (EIPA) (Fig. 1; Commisso et al. 2013). While often described as selective, EIPA works by inhibiting Na+/H+ exchangers at the plasma membrane, leading to submembranous acidification, which inhibits actin polymerization. However, given that it inhibits Na^+^/H^+^ exchangers broadly, it therefore impacts more than just macropinocytosis. Further, EIPA and other Na^+^/H^+^ exchanger inhibitors have been found to broadly affect cell growth independent of its effect on Na^+^/H^+^ exchangers (Rolver et al. 2020). Thus, there are efforts to identify alternative, more specific targets or inhibitors to perturb macropinocytosis (Lin et al. 2018; Lim et al. 2025). Notably, multiple inhibitors of VPS34 also exist, which affect both macropinocytosis and autophagy (Raufi et al. 2021). Interestingly v-ATPase, the target of bafilomycin A1, was also reported to localize to the plasma membrane, and thus also play a role in macropinosome formation (Ramirez et al. 2019). This suggests that, in addition to inhibiting lysosomal degradation of macropinocytotic cargo, bafilomycin A1 may also inhibit initiation of macropinocytosis. In some cells, macropinocytosis can be induced by decreasing glutamine availability or by treating the cells with the glutamine antagonist 6-diazo-5-oxo-L-norleucine (DON) (Lee et al. 2019b).

### Molecular assays for analyzing flux and contents of lysosomal pathways

Molecular readouts of autophagy are critical tools for any study of autophagic flux (Klionsky et al. 2021). We emphasize the importance of employing multiple, orthogonal detection methods for determining autophagic flux, as each method has a list of caveats in the guidelines that are important to consider (Klionsky et al. 2021). Additionally, recent evolutions of autophagic flux reporter systems have attempted to overcome the issue of basal RFP accumulation in mammalian cells in GFP-RFP-LC3- or RFP-GFP-LC3- based reporter systems. The more recently developed system used pulse-chase halo-tagged systems, which allows for minimal background noise and allows for time-resolved analyses of autophagic flux (Rudinskiy et al. 2022; Yim et al. 2022). From a translational perspective we believe it will be critically important to incorporate robust assays to assess autophagic flux modulation in patients, as the historical method of blotting or staining for snapshot LC3 or p62 levels may not be informative measures for target engagement of autophagy inhibitors (Tanida et al. 2005).

Methods of detecting extracellular uptake of nutrients are also widely used and are generally based on monitoring the fate of labeled extracellular materials, whether it be dextran to represent polysaccharides (Commisso et al. 2014), bovine serum albumin (BSA) to represent proteins (Marwaha and Sharma 2017), and low density lipoprotein (LDL) (Stephan and Yurachek 1993). These assays have enabled assessment not only of the degree to which cells employ micropinocytosis or receptor-mediated endocytosis, but they also allow for interrogation of conditions in which cells successfully or unsuccessfully degrade and distribute the extracellular contents.

Finally, much effort has been made to determine the molecular contents of lysosomes and autophagosomes using immunoprecipitation. By expression of tags on lysosomal protein TMEM192, one can pull-down lysosomes and further analyze their contents by using immunoblotting, or mass spectrometry-based proteomics, metabolomics, or lipidomics (Dengjel et al. 2012; Abu-Remaileh et al. 2017). Recent advances have included “tagless” lysosomal immunoprecipitation strategies based on immunoprecipitating endogenous TMEM192, which allows for isolation of lysosomes from hard-to-manipulate samples including clinical samples (Saarela et al. 2025). These innovative strategies enable dissection of the cellular and metabolic impacts of lysosomal inhibition in vivo, questions that will become increasingly relevant as novel clinical-grade lysosomal inhibitors are developed.

## Molecular outputs of lysosomal metabolism in pancreatic cancer

Numerous molecular outputs of the lysosome in PDAC have been reported, and the list continues to grow. Most hypotheses center around PDAC cells employing these pathways to maintain levels of molecular nutrients to support cellular metabolism. Others have also shown that RAS-driven tumor cells including PDAC cells employ autophagy for other functions such as maintaining mitochondrial function, redox balance, and evading antitumor immunity (Degenhardt et al. 2006; Guo et al. 2011; Yamamoto et al. 2020). Additionally, lysosome metabolism has also been shown to play a crucial role in nontumor cells contributing to the PDAC tumor microenvironment. Here, we will review the current known roles of lysosomal pathways, both tumor cell autonomous and nonautonomous, in aiding PDAC metabolism. Of note, while many of these studies were performed PDAC cell lines, many have also been shown to be conserved across different cells of origin.

### Lysosomal maintenance of iron levels through degradation of ferritin and transferrin

PDAC cells use lysosomal processes to maintain iron levels (Fig. 2). Intracellularly, PDAC cells utilize a cargo-specific form of autophagy called ferritinophagy to release iron from the iron storage protein ferritin to maintain intracellular iron homeostasis (Santana-Codina et al. 2022). In this process, nuclear receptor coactivator 4 (NCOA4) attaches itself to ferritin and delivers it to autophagosomes leading to lysosomal degradation of ferritin (Mancias et al. 2014). PDAC cells also acquire iron extracellularly through receptor-mediated endocytosis of transferrin using the transferrin receptor (TFRC) (Weber et al. 2020). Upon degradation of either ferritin or transferrin, iron (specifically ferric iron, Fe^3+^), is released and then reduced to ferrous iron (Fe^2+^) by STEAP3 (Ohgami et al. 2005). This then allows for release of iron from the lysosome to the cytoplasm by exporters DMT1/SLC11A2 (Tabuchi et al. 2000; Zhang et al. 2026) and TRPML1/MCOLN1 (Dong et al. 2008). Through these functions, the lysosome provides iron necessary for forming iron-sulfur cluster (ISC) or acting as a cofactor for proteins, such as those important for DNA replication, mitochondrial respiration, redox reactions, αKG-dependent dioxygenases, or synthesis of heme (Weber et al. 2020; Zhu et al. 2021; Santana-Codina et al. 2022; Mukhopadhyay et al. 2023; Teh et al. 2024).

**Figure 2.**
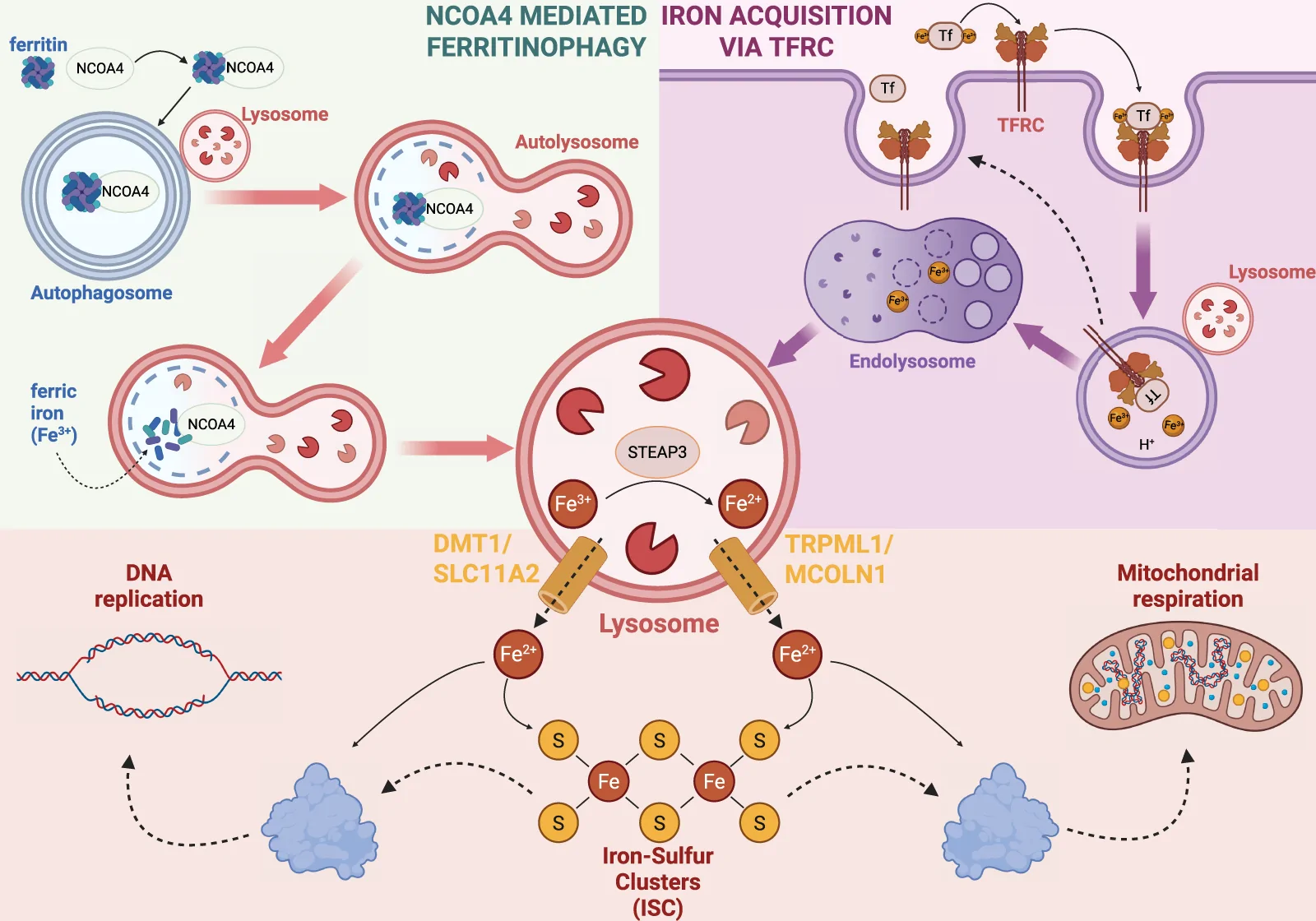
Major lysosome-dependent iron metabolism pathways. Intracellular iron is stored as ferritin, which can be degraded through NCOA4-dependent ferritinophagy, releasing free iron. Extracellular iron can be acquired when bound to transferrin through transferrin receptor (TFRC)-mediated endocytosis. The acidic environment of the lysosome stimulates release of (ferric) iron from these carrier molecules. It is then reduced by STEAP3 and released to the cytoplasm, where it contributes to iron-sulfur clusters. These, along with iron itself, serve as cofactors for cellular enzymes important for functions such as DNA replication and mitochondrial respiration.

Thus, targeting iron metabolism through or in conjunction with targeting lysosomal functions has been proposed as a therapeutic strategy. NCOA4 was found to be overexpressed in human PDAC cells compared to matched normal pancreas cells. Additionally, a low ferritinophagy gene expression signature was reported to correlate with better prognosis in PDAC patient data sets. Together, these data suggest that PDAC cells employ ferritinophagy at an elevated rate. Furthermore, NCOA4 ablation delays tumor progression and increases survival in preclinical PDAC models (Santana-Codina et al. 2022). Similarly, disruption of autophagy or the lysosome has been shown to decrease PDAC cell proliferation and tumor growth in an iron-dependent manner (Weber et al. 2020; Mukhopadhyay et al. 2023).

In our opinion, there are three important nuances to address regarding the strategy of depleting PDAC cellular iron by targeting the autophagy/lysosomal system. First, while acquiring enough iron to sustain these functions is a critical role of the lysosome through these pathways, high ferritinophagic flux also makes PDAC cells vulnerable to an iron-dependent form of cell death termed ferroptosis (Hou et al. 2016). Consistent with this, overexpression of NCOA4 promoted ferroptosis, while knockdown of NCOA4 suppressed ferroptosis (Hou et al. 2016). Thus, activating ferritinophagy might enable leveraging ferroptosis as a therapeutic inroad for PDAC.

Second, PDAC cancer associated fibroblasts (CAFs) can support PDAC iron levels in response to IL-6 signaling. Thus, to most potently deplete PDAC cellular iron, the tumor microenvironmental iron levels must also be considered. As a proof of concept, a recent study demonstrated that placing mice on low-iron diets enhanced the effects of autophagy or lysosomal inhibition on PDAC tumor growth (Mukhopadhyay et al. 2023).

Finally, while lysosomal inhibition leads to decreased available iron, it seems that not all lysosomal inhibitors affect cell growth specifically through decreasing available iron. For example, while multiple reports demonstrate that iron supplementation rescues cells from treatment with bafilomycin A1, there have been mixed reports on the ability of iron supplementation to rescue cells from treatment with chloroquine (Devenport et al. 2021; Mukhopadhyay et al. 2023; Cheng et al. 2025) or PIKfyve inhibitors (Cheng et al. 2025; Cao et al. 2026). One possible explanation to explain these differences centers on the role of pH in the release of ferric iron from ferritin (Asano et al. 2011) and transferrin (Mazurier and Spik 1980), as well as affecting the activity of enzymes and transporters such as MCOLN1 (Dong et al. 2008). These notions support the concept that lysosomal pH is specifically necessary for lysosomal iron metabolism (Yambire et al. 2019; Weber et al. 2020); however, it would imply that bafilomycin A1, chloroquine, and PIKfyve inhibitors do not affect lysosomal pH to the same degree. While this has been shown to be true in some instances (Mauthe et al. 2018; Ponsford et al. 2021; Leray et al. 2022), this hypothesis is only based on correlational observations, and it is unclear whether this truly leads to how these different inhibitors affect whether they affect cell growth through disrupting iron metabolism. Future studies are warranted to describe the apparent discrepancies between these inhibitors and their effects cell lysosomal iron metabolism.

### Lysosomal maintenance of amino acid levels and nucleotide metabolism

Lysosomal processes have well-documented roles in driving nucleotide and amino acid metabolism in PDAC (Fig. 3). Most notably, PDAC macropinocytosis permits that acquisition of extracellular proteins that can then contribute to amino acid and nucleotide metabolism (Kamphorst et al. 2015; Zhang and Commisso 2019).

**Figure 3.**
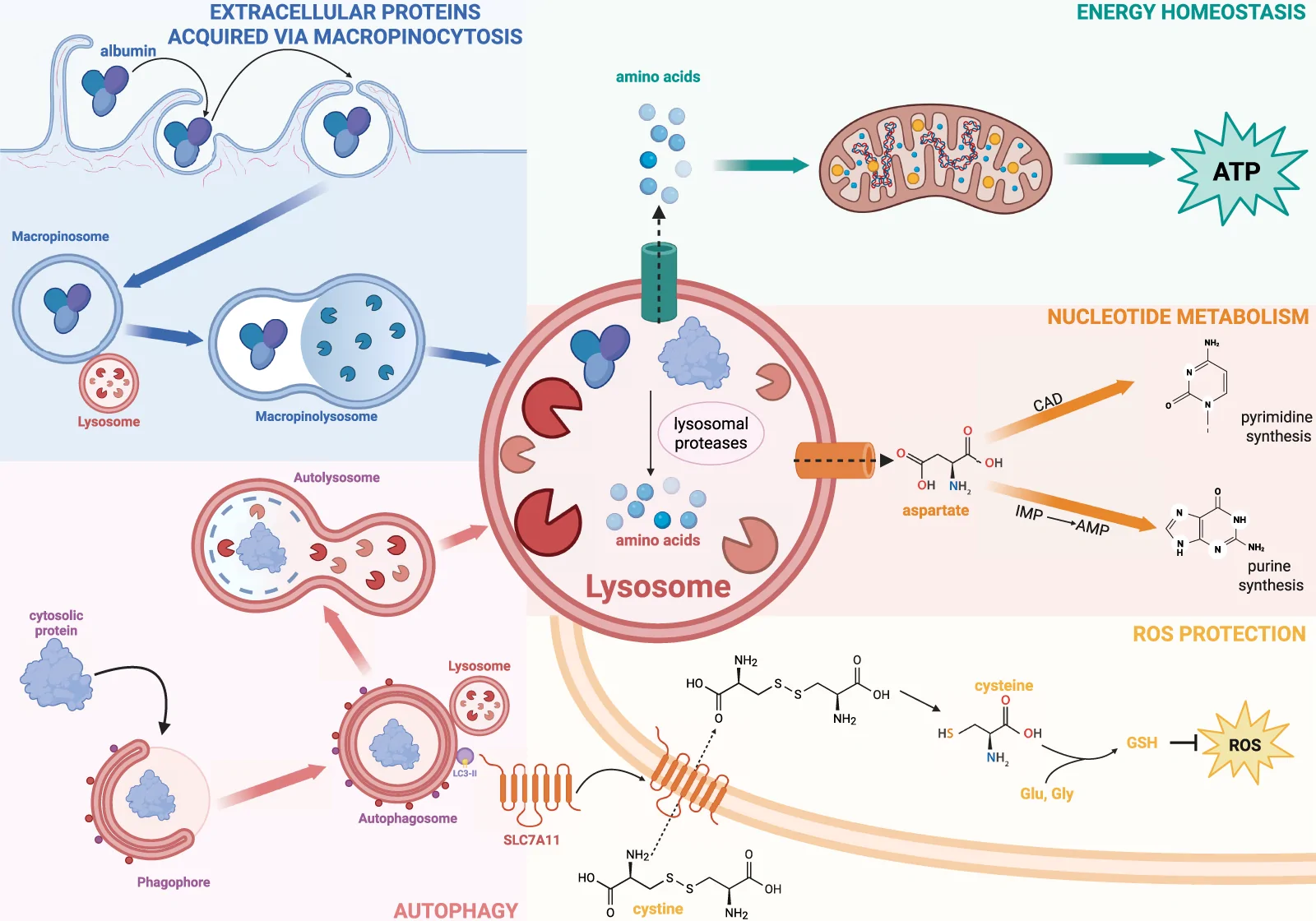
Major lysosomal pathways supporting amino acid and nucleotide metabolism. Macropinocytosis is a major source of protein uptake and amino acid acquisition. Autophagy degrades unused/superfluous proteins and also allows for cystine transporter SLC7A11 to translocate to the plasma membrane. Generated amino acids contribute to central carbon metabolism and energy supply, nucleotide pools. SLC7A11 imports cystine which can eventually be used for glutathione synthesis and ROS protection.

Numerous studies of autophagy inhibition in PDAC cells have also demonstrated a crucial role of autophagy in regulating amino acid metabolism. For example, knockdown of TFE3 or ATG5 or treatment with bafilomycin A1 led to a preferential decrease in amino acid levels specifically in PDAC cell lines and not in normal pancreas cells (Perera et al. 2015). Further, starvation of autophagy-deficient (ATG7 knockout) cells can be fully rescued by addition of glutamine or glutamate, emphasizing the importance of autophagy in maintaining levels of amino acids (Guo et al. 2016). Other studies have pointed to autophagy as having a role in regulating cysteine metabolism through controlling regulation of the cystine importer SLC7A11 (Mukhopadhyay et al. 2021). Finally, one other study indicated that chloroquine treatment depleted PDAC cells of aspartate (Elliott et al. 2019).

Notably, while amino acids can be utilized in many ways in the cell, protection from reactive oxygen species (ROS) and maintaining nucleotide levels have been recurrently reported as the fates of the amino acids derived from lysosomal metabolism. For example, cystine imported from SLC7A11 activity was hypothesized to contribute to cysteine availability and thus ROS protection. This is supported by the findings that *N*-acetylcysteine (NAC) protected PDAC cells from autophagy or lysosome inhibition (Mukhopadhyay et al. 2021). An independent study suggested from stable isotope tracing data that glutamine may contribute to generating NADPH in autophagy-deficient cells, a well-known phenomenon of PDAC cells (Son et al. 2013). However, in this study, while NAC indeed decreased starvation-induced ROS in autophagy-deficient cells, it did not rescue proliferation defects (Guo et al. 2016). Instead, this study pointed to the role of autophagy in maintaining energy homeostasis (triphosphate nucleotides) and overall nucleotide pools. In line with this, an aforementioned study pointed to the effects of lysosome inhibition on limiting de novo nucleotide biosynthesis due to aspartate depletion leading to replication stress (Elliott et al. 2019). Finally, recent data from a study on PDAC cells that are resistant to autophagy or lysosome inhibition indicated that these cells have lower levels of pyrimidines and lower levels of pyrimidine synthesis, making them more dependent on pyrimidine salvage (Dufresne et al. 2025).

Together, these studies highlight the role that macropinocytosis and autophagy play in obtaining amino acids to maintain metabolic homeostasis through functions such as protection from ROS, maintenance of energy charge, and contributing to nucleotide pools.

### Lysosomal regulation of lipid homeostasis

While it is well known that lysosomes play an indispensable role in regulating lipids (Ebner et al. 2025), the precise mechanisms through which PDAC employs this metabolism is less well defined (Fig. 4). In our recent study, we performed a metabolism-wide CRISPR screen in PDAC cells and determined that sphingolipid and cholesterol synthesis pathways were among the most selectively essential metabolic functions upon PIKfyve inhibition. Importantly, PIKfyve inhibition and other forms of lysosome inhibition (i.e., chloroquine and bafilomycin A1 treatment), potently activated a lipogenic transcriptional program, suggesting that PDAC cells have an increased utilization and dependency on sphingolipid and cholesterol synthesis when the lysosome is inhibited (Cheng et al. 2025). These results strongly corroborated data obtained from previous metabolic CRISPR screens performed in other cell types, also highlighting cholesterol and sphingolipid synthesis as liabilities when lysosomes are inhibited (Weber et al. 2020; Unlu et al. 2022). Further, the finding that PIKfyve inhibition disrupts lipid metabolism has been subsequently reported in other cancer types by our groups and others, further emphasizing the presence of a generalizable functional connection between lysosome and lipid synthesis in cancer cells (Bonolo De Campos et al. 2025; Cao et al. 2026; Zheng et al. 2026). However, to our understanding, the source of these lipids and their functional roles are still unclear.

**Figure 4.**
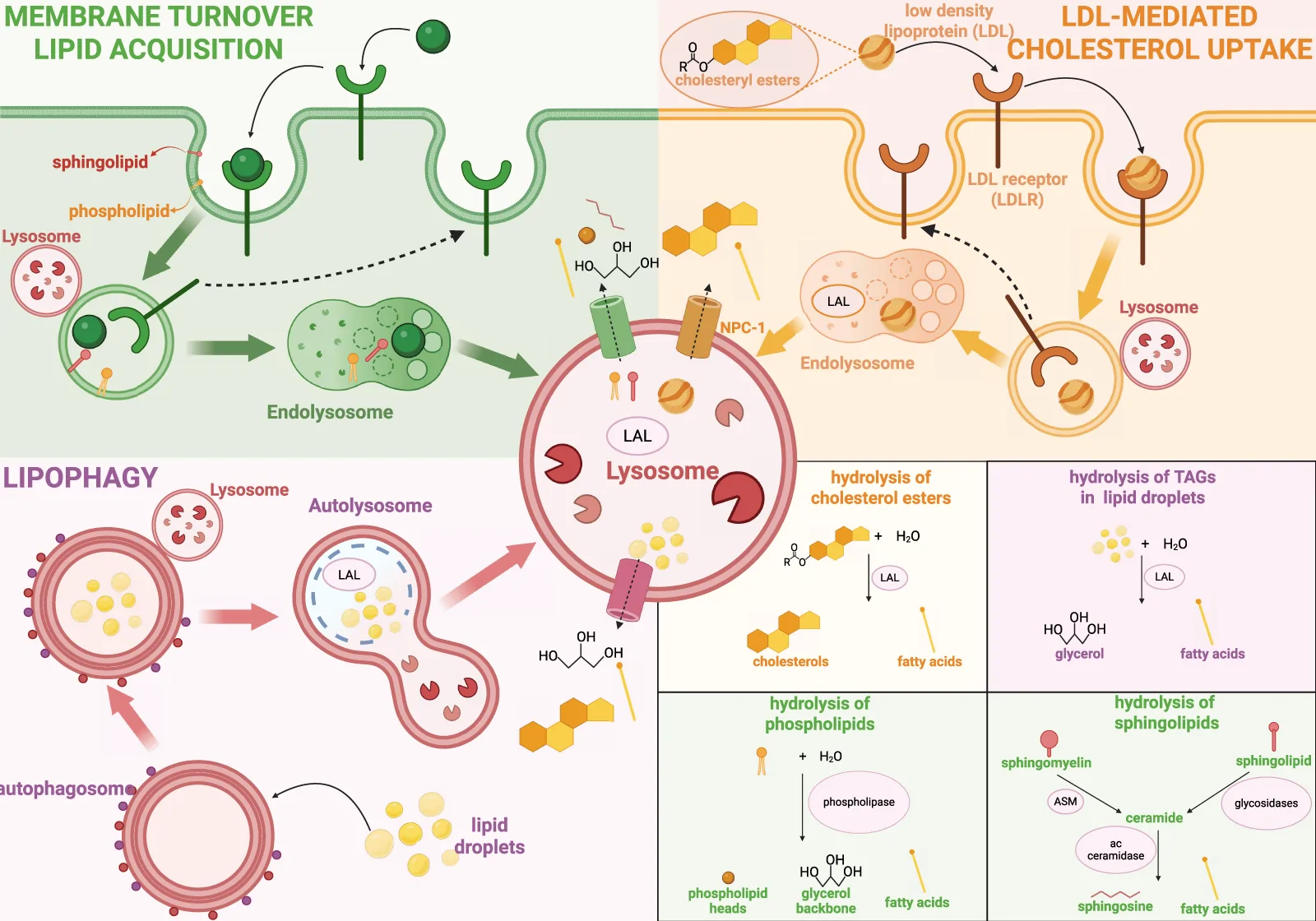
Major lysosomal pathways supporting lipid metabolism. (*Top left*) Membrane lipids, notably sphingolipids, undergo turnover often through endocytosis resulting in lipid breakdown and reshuffling. (*Top right*) Cholesterol esters carried by LDL can be internalized through LDL receptor-mediated endocytosis. (*Bottom left*) Lipid droplets containing mainly triglycerides and cholesterol esters can be degraded by lipophagy. The lysosome contains numerous lipases that aid in breaking down or hydrolyzing lipids to base macromolecules that can be utilized after exported to the cytoplasm.

Lipophagy is cargo-specific form of autophagy carried out in the lysosome that serves as an alternative pathway for degrading lipid droplets, as opposed to lipolysis, which occurs in the cytosol (Singh et al. 2009). These lipid droplets allow the cell to store lipids in the form of triglycerides or cholesterol esters when energy supplies are high, and lipophagy and lipolysis allow the cell to mobilize such lipids when needed (Maan et al. 2018). While the role lipophagy plays in driving PDAC is still unclear, a recent report demonstrated that inhibiting lipophagy led to increased lipid droplet accumulation, which disrupted fatty acid metabolism and suppressed cell growth (Zhang et al. 2024). On the other hand, blocking cholesterol esterification and thus lipid droplet production was also reported to suppress PDAC growth (Li et al. 2016; Oni et al. 2020), suggesting that the control of storage and utilization of lipids and lipid droplets may be important for PDAC metabolic homeostasis.

PDAC cells uptake extracellular lipids in the form of LDL through LDLR-mediated endocytosis. Upon internalization, LDL is degraded in the lysosome, releasing the lipids (mostly cholesterol, some triglycerides) which can then be transported out into the cytosol through receptors such as NPC1 (Ebner et al. 2025). *LDLR* was found to be overexpressed in a mouse PDAC GEMM (LSL-Kras^G12D^; Ink4a/Arf^fl/fl^; and Pdx1-Cre) versus control (LSL-Kras^G12D^; Ink4a/Arf^fl/fl^), which led to increased levels of cholesterol in PDAC vs control cells. In line with this, blocking the ability of PDAC cells to uptake lipids through LDLR knockdown decreased cell growth and potentiated chemotherapy (Guillaumond et al. 2015), suggesting that PDAC cells rely in part on this pathway to obtain lipids necessary for proliferation. Interestingly, PCSK9, which promotes degradation of LDLR (thus negatively regulating cholesterol uptake) was found to influence PDAC metastatic site, in that PCSK9-low tumors metastasized to the LDL-rich liver, while PCSK9-high tumors metastasized to LDL-poor lung (Rademaker et al. 2025). These studies suggest that cholesterol uptake plays a role in PDAC tumor growth and metastasis and also highlight an example of host metabolism interacting with tumor lysosomal processes.

Lysosomes are also known to play a role in cell membrane turnover. In fact, some lysosomal storage diseases are caused by the inability of the lysosome to degrade sphingolipids, mostly originating from the plasma membrane and delivered to the lysosome through endocytosis (Jeyakumar et al. 2002). Additionally, lysosomes have also been shown to degrade membrane phospholipids when de novo lipid synthesis is blocked in an autophagy-independent manner (Hosios et al. 2022). To our knowledge, it has not been explicitly shown whether PDAC utilizes lysosomes to catabolize cell membranes for lipid remodeling. However, given that CRISPR screens with the selective pressure of lysosome inhibition recurrently highlight sphingolipid synthesis as a synthetic lethality, we suspect that it may be a functionally relevant role of lysosomal metabolism in PDAC and other cancer cells (Unlu et al. 2022; Cheng et al. 2025). Advancements in mass spectrometry- based lipidomics and isotope tracing, lysosomal immunoprecipitation, and lipid probes (Altuzar et al. 2023) may allow for more precise assessments of this pathway.

### Lysosomal contribution to immune evasion

Multiple reports have identified that autophagy enables immune evasion in PDAC cells. Specifically, autophagy inhibition was found to enhance T-cell mediated killing of PDAC cells resulting in restrained tumor growth in immune competent models while having no effect on tumor growth in immune deficient models. However, while some reports suggest that inhibition of autophagy blocks autophagic degradation of MHC1 resulting in increased MHC1 presentation (Yamamoto et al. 2020; Bao et al. 2023), others have reported that autophagy inhibition sensitizes PDAC cells to TNFα secreted by T cells (Zhu et al. 2021). The clarification of this mechanism is an important topic of future studies.

### Lysosomal pathways in nontumor cells in the PDAC tumor microenvironment

While many studies have demonstrated that lysosomal metabolism plays an important role in tumor cells, a growing body of literature also demonstrates the importance of these pathways in cells in the tumor microenvironment as well as the entire host. Specifically, pancreatic stellate cells in the tumor microenvironment have been shown to provide amino acids such as alanine through autophagic degradation of proteins upon stimulation by PDAC cells (Sousa et al. 2016). Furthermore, pancreatic stellate cell activation and transition into cancer associated fibroblasts (CAFs) is dependent on autophagy (Endo et al. 2017), as is CAF collagen production (Bai et al. 2023). These mechanisms underly the phenotypic findings that autophagy ablation in stellate cells, CAFs, or the whole host lead to decreased tumor growth, even while the tumor cells remain autophagy-competent (Sousa et al. 2016; Endo et al. 2017; Bai et al. 2023). In addition to autophagy, CAFs, specifically myofibroblasts (myCAFs), employ macropinocytosis under conditions of nutrient (i.e., glutamine) stress (Zhang et al. 2025). This process was shown to support CAF fitness, collagen production, as well as maintenance of amino acid pools to support PDAC tumor cells (Zhang et al. 2021).

## The current clinical landscape of targeting lysosomal pathways in PDAC

Targeting lysosomal pathways for PDAC represents a promising strategy conceptually, in that they support not only metabolic homeostasis but also allow for immune evasion in PDAC tumors (Yamamoto et al. 2020; Zhu et al. 2021). However, in clinical settings inhibitors of lysosomal pathways have not yet produced favorable results in PDAC (Table 1). In this section, we will review the efforts to target this pathway as PDAC therapy, both as single agents and in combination with other therapies, with a focus on its preclinical and clinical efficacy.

**Table 1.** List of past and current clinical trials testing autophagy or lysosome inhibitors in pancreatic cancers

Drug	Cancer type/stage	Phase	Status	Result	Notes	Citation
HCQ	Metastatic PDAC (previously treated)	II	Completed	Insufficient efficacy	Inconsistent autophagy inhibition (PBMC LC3-II levels)	Wolpin et al. 2014 NCT01273805
Gemcitabine ± HCQ	Preoperative PDAC	I/II	Completed	Safe and well-tolerated. Patients with evidence of autophagy inhibition had improved survival	Autophagy inhibition measured by PBMC LC3-II	Boone et al. 2015 NCT01128296
Gemcitabine + nab-paclitaxel ± HCQ	Metastatic PDAC (1L)	II	Completed	Improved response rate, but no change in survival	Adjuvant chemotherapy or radiotherapy was allowed if administered at least 4 months prior	Karasic et al. 2019 NCT01506973
Gemcitabine + nab-paclitaxel ± HCQ	Preoperative PDAC	I/II	Completed	Safe and well tolerated, greater pathological response, improved serum biomarker (CA19-9) response, evidence of autophagy inhibition and immune activity. No difference in survival	Autophagy inhibition measured by cytoplasmic p62/SQSTM1 staining. No change in LC3-II	Zeh et al. 2020 NCT01978184
LY32114996 ± HCQ	Metastatic PDAC (2L/3L)	II	Completed	No change in survival.		Surana et al. 2025 NCT04386057
Binimetinib ± HCQ	Metastatic PDAC (previously treated)	I	Completed	Challenging toxicity profile; minimal clinical efficacy observed		Haldar et al. 2026 NCT04132505
DCC3116 ± trametinib	Metastatic solid tumors with RAS or RAF mutations	I/II	Active, not recruiting	No results posted	Abstract	NCT04892017
ESK981	Advanced solid tumors: PDAC, GEP-NET, NEPC	II	Recruiting	No results posted		NCT05988918

Hydroxychloroquine (the safer, less toxic derivative of chloroquine [Cabral et al. 2019]), is the most extensively studied and utilized lysosome inhibitor (Table 1). It is an FDA-approved drug for the treatment of malaria, rheumatoid arthritis, and systemic lupus erythematous. In the case of malaria, (hydroxy)chloroquine binds to hemozoin, the growing polymer chain of heme resulting from hemoglobin degraded in the food vacuoles of malarial parasites. (Hydroxy)chloroquine binding to hemozoin caps polymerization and thus reduces available nutrient sources for the parasites, while also leading to accumulation of heme that is toxic to the parasites (Sullivan et al. 1996). In the case of rheumatoid arthritis and systemic lupus erythematous, (hydroxy)chloroquine's mechanism of action is still under investigation and may be context-dependent (Schrezenmeier and Dorner 2020). For example, while it has been shown to inhibit cytokine production and modulate co-stimulatory molecules in part through interfering with lysosomal activity and autophagy, it has also been shown to target a number of other nonlysosomal or autophagy-related targets. Given its historical clinical utilization and its property as a lysosomotropic compound, it was a logical candidate to test if human PDAC depend on lysosomal activities. However, as a single agent, it was unable to reliably inhibit autophagy or noticeably control tumor growth at the same doses in a phase II clinical trial in PDAC patients (NCT01273805) (Wolpin et al. 2014).

Retrospectively, these results could be considered consistent with preclinical data, which showed that chloroquine had inconsistent efficacy in various tumor models as a single agent. For example, in PaTu-8988T-derived subcutaneous xenografts in nude mice, chloroquine treatment dramatically reduced tumor growth, even leading to complete regressions in a subset of tumors (Yang et al. 2011). However, in a separate study, chloroquine treatment had nearly unappreciable effects as a single agent in subcutaneous tumors generated from four different cell lines or patient-derived xenografts in NOD/SCID mice (Kinsey et al. 2019). Similarly, chloroquine therapy had insignificant effects on end point tumor volume in a syngeneic orthotopic tumor model (Yamamoto et al. 2020). These studies highlighted the importance of robustly demonstrating preclinical efficacy in a multitude of models, particularly ones that better represent physiological conditions (such as orthotopic and spontaneous models).

One strategy for incorporating lysosome inhibitors into PDAC therapy was in combination with chemotherapy. This was clinically straightforward to test given that chemotherapy is first line treatment for PDAC. Preclinically, chemotherapy has been shown to increase autophagic flux in PDAC cells, and the combination of chemotherapy with chloroquine has been shown to increase cytotoxicity and tumor growth control in PDAC models (Fu et al. 2018). Most recently, mechanistic rationale supporting the combination autophagy inhibitors and chemotherapeutic agents was proposed. Specifically, this study demonstrated that PDAC cells resistant to chloroquine or ULK inhibitor MRT68921 had lower rates of pyrimidine synthesis and increased rates of pyrimidine salvage exposing them to pyrimidine analog-based chemotherapies such as gemcitabine (Dufresne et al. 2025).

Early clinical trials indicated that patients who had successful autophagy inhibition (as measured by increased LC3 staining in peripheral blood mononuclear cells, PBMCs) upon neoadjuvant hydroxychloroquine and gemcitabine treatment had improved survival outcomes in patients with potentially resectable tumors (NCT01128296) (Boone et al. 2015). However, in a follow-up study in patients at a similar stage, the addition of hydroxychloroquine to chemotherapy did not improve survival outcomes (NCT01978184) (Zeh et al. 2020). Of note, these studies did find that the addition of hydroxychloroquine increased tumor immune infiltration, improved tumor pathologic response, and decreased serum CA 19-9 levels. They also found an increased level of p62/SQSTM1 but not LC3 staining with the addition of hydroxychloroquine in samples of the resected tumors. Another clinical trial assessing the effects of hydroxychloroquine along with chemotherapies in metastatic PDAC patients demonstrated that the addition of hydroxychloroquine to standard therapy improved response rates but did not result in improved survival in PDAC patients (NCT01506973) (Karasic et al. 2019). Of note, in these studies, hydroxychloroquine was administered at an elevated dose of 600 mg twice daily, as opposed to the standard dose (200–400 mg daily) given for other diseases. Taking all of the clinical trial results together, (hydroxy)chloroquine has not contributed to improvement in pancreatic patient outcomes as a single agent or in combination with chemotherapy (Table 1). These studies all point to the potential role that autophagy or lysosome inhibitors have in PDAC; however, it was questionable whether hydroxychloroquine was able to effectively inhibit autophagy. Additionally, these studies also emphasize the need to develop a practical and reliable method for detecting changes in autophagic flux in patients enrolled in clinical trials.

PDAC harbor near ubiquitous mutations in KRAS, along with recurrent losses of tumor suppressors, such as p53, p16 (CDKN2A), and SMAD4 (Waddell et al. 2015). The mutations in KRAS lead to overactive KRAS signaling through the MAPK pathway, which serves as the main oncogenic driver in PDAC (Klomp et al. 2024a,b). Thus, there has been an enormous effort to develop therapeutics targeting this pathway (Hitchen et al. 2025). A series of studies in 2019 found that inhibition of KRAS–MAPK forced PDAC cells to increase utilization and dependence on autophagic flux (Bryant et al. 2019; Kinsey et al. 2019; Lee et al. 2019a). This revelation challenged previous hypotheses that assumed KRAS–MAPK drove autophagic flux given that it drives many metabolic pathways in PDAC. These studies also established the concept of dual targeting KRAS–MAPK and autophagy using (hydroxy)chloroquine, as they showed that this strategy resulted in synergistic effects in in vitro and in vivo preclinical models as well as in a PDAC patient through compassionate use (Bryant et al. 2019; Kinsey et al. 2019). Additionally, multiple additional case reports have demonstrated clinical benefit from the combination treatment of trametinib, a MEK inhibitor, and hydroxychloroquine in metastatic PDAC (Xavier et al. 2021; Wu et al. 2022). Given promising preclinical results, this regimen was tested in cancer centers in Germany, and formal clinical trials were also launched assessing the efficacy of ERK inhibitor LY3214996 (NCT04386057) or BRAF inhibitor binimetinib (NCT04132505) in combination with hydroxychloroquine in the United States. However, a retrospective study on the efforts in Germany reported that no clinical benefit was observed with the combination of trametinib and hydroxychloroquine (Witte et al. 2024). Additionally, the combination of hydroxychloroquine and LYS3214996 also did not result in consistent autophagy inhibition or clinically meaningful outcome benefits (Surana et al. 2025). Finally, the combination of binimetinib and hydroxychloroquine was shown to have limited clinical efficacy, mainly limited by a challenging toxicity profile (Halder et al. 2026). These results collectively suggest that targeting MEK or ERK in conjunction with using hydroxychloroquine is not an effective combination therapy for PDAC.

Although the completed clinical trials indicate that hydroxychloroquine treatment does not improve survival outcomes, we believe that these studies still give reason to continue pursuing targeting these pathways clinically and more importantly highlight important lessons to be learned. First, early indications suggested that patients who had successful autophagy inhibition had significantly improved survival outcomes. Subsequent studies also indicated that, while overall survival metrics were not significantly changed, response rates were improved with hydroxychloroquine treatment. Of note, the studies that assessed tumor autophagy inhibition indicated that hydroxychloroquine had inconsistent inhibition of autophagy in tumors and in PBMCs (Zeh et al. 2020; Surana et al. 2025). Given this, there remains considerable activity around identifying superior targets and compounds to inhibit lysosomal pathways for PDAC therapy. Some example compounds include the ULK1/2 inhibitor DCC3116 (NCT04892017) (Tolcher et al. 2022), as well as PIKfyve inhibitors apilimod and ESK981.

PIKfyve is the lysosomal target most actively studied as a clinical target, as there are two PIKfyve small molecule inhibitors that have cleared phase I clinical trials: apilimod (NCT02594384) (Harb et al. 2017; Diefenbach et al. 2020) and ESK981 (NCT00875264) (Pili et al. 2014). As previously mentioned, while apilimod is safe and specific for PIKfyve, it has poor pharmacokinetic properties, precluding it from utilization in the context of cancer therapies (Ikonomov et al. 2019). ESK981 was initially developed as a multikinase inhibitor targeting VEGFR and cleared phase I clinical trials in patients with advanced cancers (Pili et al. 2014). However, our laboratory later identified PIKfyve as the target of ESK981, which suppressed tumor growth and potentiated immune checkpoint therapy in prostate cancer models (Qiao et al. 2021). Unfortunately, phase II clinical trials assessing the efficacy ESK981 as a single agent or with nivolumab showed no anti-tumor activity in metastatic castration resistant prostate cancer (NCT03456804 [Heath et al. 2024b], NCT04159896 [Heath et al. 2024a]).

In PDAC, a phase II clinical trial assessing ESK981 as a single agent in select solid tumors including PDAC is currently ongoing (NCT05988918) given our preclinical data that demonstrated ESK981 having much more potent anti-tumor efficacy in PDAC (Cheng et al. 2025) than AR-positive prostate cancer. Additionally, we and others have shown that PIKfyve inhibition synergizes with RAS-MAPK inhibitors in preclinical models (Cheng et al. 2025; DeLiberty et al. 2025). Given the recent breakthroughs of the development of novel RAS-MAPK inhibitors, we hope to test the efficacy of adding ESK981 to RAS-MAPK therapy in PDAC clinical trials.

## Impacts of autophagy on diet, health, and cancer

Autophagy has recently become a topic of conversation in nonscientific fields, often in the context of caloric restriction, (intermittent) fasting, and longevity. Here, caloric restriction leads to the induction of autophagy to maintain organismal metabolic homeostasis. There is strong evidence from human studies, including population studies and randomized control trials, that caloric restriction without malnutrition results in health benefits (Most et al. 2017; Dorling et al. 2021). These include increased insulin sensitivity (Larson-Meyer et al. 2006; Weiss et al. 2006; Johnson et al. 2016), cardiovascular health (Lefevre et al. 2009; Kraus et al. 2019), decreased inflammation (Fontana et al. 2004; Ravussin et al. 2015) while maintaining or improving immune function (Ahmed et al. 2009; Meydani et al. 2016), decreased oxidative damage (Hofer et al. 2008), and a host of other physiological and psychological effects. Notably, while there is still not causative, long-term data strictly on the relationship between caloric restriction and mortality or longevity in humans, the aforementioned biomarkers are strongly correlated with health span (or inversely correlated with morbidity and mortality).

In line with this, diet and nutrition also have close ties to the development of cancer. Preclinical models have demonstrated that obesity can promote pancreatic cancer growth, and that weight normalization can slow obesity-accelerated pancreatic cancer development (Wirkus et al. 2025). In other preclinical models, caloric restriction (regardless of dietary strategy) was shown to slow tumor growth (Pomatto-Watson et al. 2021). However, it is important to note that while short-term fasting did indeed decrease tumor growth, eventual re-feeding led some tumors to grow and attain similar sizes to those in animals on the control diet (Lee et al. 2012). Additionally, fasting has been shown to reshape the tumor metabolic microenvironment to boost anti-tumor immunity in preclinical models (Delconte et al. 2024). Although these concepts have not been tested in randomized clinical trials, they are strongly in line with observational studies mentioned previously about the benefits on caloric restriction.

## Conclusions and future perspective: leveraging nutritional availability and lysosomal nutrient acquisition and recycling pathways in cancer therapy

Autophagy has been shown to promote health and longevity and suppresses tumor development; yet, once tumors have formed, autophagy reciprocally sustains cancers, such as pancreatic cancer. This tension highlights the importance of considering the contexts in which it would be beneficial to promote or inhibit autophagy systemically (White 2015; Chavez-Dominguez et al. 2020). Of note, many of these conclusions are mere speculations based on mostly preclinical data; they are not intended to inform medical or personal decision-making. Robust, large-scale randomized control trials will be necessary to make real-world recommendations.

It is currently unclear whether activating or inhibiting autophagy is beneficial in the setting of undetectable tumor burden. Of course, activating autophagy provides a host of benefits in healthy individuals as described above. Autophagy has also been shown to suppress tumor development by limiting DNA damage and chromosomal instability (Mathew et al. 2007), in line with the work cited above related to caloric restriction and cancer development. However, empirical evidence has shown that blockade of autophagy has a p53-dependent effect on tumor initiation, in that autophagy blockade (*Atg5* knockout) in the setting of heterozygous loss of p53 inhibited tumor formation (Yang et al. 2014), while autophagy blockade (*Atg7* or *Atg5* knockout, or chloroquine treatment) in the setting of homozygous loss of p53 promoted tumor formation (Rosenfeldt et al. 2013). In line with this, our recent data suggested that *Pikfyve* deletion or inhibition delayed tumor formation in PDAC models harboring heterozygous p53 loss (Cheng et al. 2025). These uncertainties warrant further study, particularly into whether promoting or inhibiting whole-body autophagy is protective against tumor development.

For patients receiving chemotherapy and/or immunotherapy, activating systemic autophagy with fasting/fasting mimicking diets have been shown to potentiate therapy while protecting healthy cells (Nencioni et al. 2018). The mechanistic hypothesis, known as the differential stress response, is that starvation induces a reduction in proliferation of normal cells and increases protective autophagy and other surveillance mechanisms, making them less susceptible to chemotherapy. In contrast, cancer cells do not respond to growth inhibitory signals due to being stuck in perpetual oncogenic signaling. As such, these cells retain sensitivity to chemotherapy, thereby increasing the therapeutic window (Raffaghello et al. 2008). The increased efficacy of chemotherapy in fasting subject has also be shown to partially mediated by enhanced antitumor immunity (Di Biase et al. 2016; Pietrocola et al. 2016), which can be further leveraged with immunotherapy (Ajona et al. 2020). While combining fasting with therapy resulted in even more potent tumor suppressive efficacy in preclinical models, quality of life and tolerability concerns are important to consider in the attempt to apply these strategies to clinical settings (Caccialanza et al. 2019; Blaževitš et al. 2023; Pio et al. 2025). Early trials have promisingly demonstrated that fasting-mimicking diet is tolerable, changes the tumor metabolic and immunologic landscape, and enhances chemotherapy in 101 patients with different tumor types (Ligorio et al. 2022; Vernieri et al. 2022). However, large-scale clinical studies will be necessary to determine the contexts in which these strategies can be applied.

Several questions about the utility of targeting autophagy and lysosomal nutrient acquisition/recycling pathways remain. First, there continues to be a longstanding need for the development of more potent clinical-grade autophagy or lysosome inhibitors. As mentioned previously, there is no shortage of preclinical compounds that inhibit autophagy or lysosomes; however, to our knowledge, only hydroxychloroquine, apilimod, ESK981, and DCC3116 have been tested clinically, none of which demonstrated single agent efficacy. The continued discovery and validation of favorable drug targets will hopefully aid in the eventual development of a safe, potent autophagy/lysosome inhibitors. In parallel, adopting accurate and scalable measurements of autophagic flux (Hein et al. 2026), as opposed to a snapshot level of autophagic markers (which may not accurately reflect autophagic activity [Tanida et al. 2005]), will aid in more robust pharmacodynamic evaluations in clinical trials.

Second, it will be important to clarify whether targeting autophagy specifically or targeting lysosomes themselves is the optimal strategy. This is especially critical given the multiple reports that the essentiality of autophagy is heavily context-dependent. We speculate that inhibiting lysosomes would effect a more comprehensive metabolic deprivation; however, it may also lead to increased toxicity to other cells in the body. Ongoing work is also demonstrating the possible benefit to inhibiting both an upstream autophagy mediator as well as the lysosomes, which could fine-tune the efficacy/toxicity balance should that exist, or achieve a more complete blockade of autophagic flux (Roach et al. 2024).

Finally, it is unlikely that autophagy or lysosome inhibition alone will be sufficient for eliciting a clinically significant decrease in tumor growth. Preclinical studies have already demonstrated tumor intrinsic and extrinsic resistance mechanisms to autophagy and lysosome inhibition (Mukhopadhyay et al. 2023; Cheng et al. 2025; Dufresne et al. 2025). Thus, further identification of synthetic dependencies upon autophagy/lysosome inhibition may provide new avenues to develop combination therapy or even dietary modification (Mukhopadhyay et al. 2023) strategies to enhance the efficacy autophagy/lysosome inhibitors. Perhaps the most promising and likely translatable strategy will be combining autophagy/lysosome inhibitors with the new mutant-specific KRAS, pan-KRAS, or pan-RAS inhibitors. We believe that the mechanistic rationales and the demonstrated preclinical efficacy shown by the combination of autophagy/lysosome inhibitors with KRAS-MAPK inhibitors will apply to these next-generation RAS inhibitors and enhance the already unprecedented effects they have shown in clinical trials. (Bryant et al. 2019; Kinsey et al. 2019; Lee et al. 2019a; Cheng et al. 2025; DeLiberty et al. 2025).
